# Inactive alleles of cytochrome P450 *2C19* may be positively selected in human evolution

**DOI:** 10.1186/1471-2148-14-71

**Published:** 2014-04-01

**Authors:** Ramatoulie E Janha, Archibald Worwui, Kenneth J Linton, Seif O Shaheen, Fatoumatta Sisay-Joof, Robert T Walton

**Affiliations:** 1 Medical Research Council Unit The Gambia, The Gambia, West Africa; 2 Centre for Public Health and Primary Care, Barts and the London School of Medicine and Dentistry, Queen Mary University, London, England; 3 Centre for Cutaneous Research, Blizard Institute, Barts and the London School of Medicine and Dentistry, Queen Mary University, London, England

**Keywords:** Positive selection, Cytochrome P450 2C19, Xenobiotics, Drug metabolism, Extended haplotype homozygosity, Bifurcation plots

## Abstract

**Background:**

Cytochrome P450 CYP2C19 metabolizes a wide range of pharmacologically active substances and a relatively small number of naturally occurring environmental toxins. Poor activity alleles of *CYP2C19* are very frequent worldwide, particularly in Asia, raising the possibility that reduced metabolism could be advantageous in some circumstances. The evolutionary selective forces acting on this gene have not previously been investigated.

We analyzed *CYP2C19* genetic markers from 127 Gambians and on 120 chromosomes from Yoruba, Europeans and Asians (Japanese + Han Chinese) in the Hapmap database. Haplotype breakdown was explored using bifurcation plots and relative extended haplotype homozygosity (REHH). Allele frequency differentiation across populations was estimated using the fixation index (F_ST_) and haplotype diversity with coalescent models.

**Results:**

Bifurcation plots suggested conservation of alleles conferring slow metabolism (CYP2C19*2 and *3). REHH was high around CYP2C19*2 in Yoruba (REHH 8.3, at 133.3 kb from the core) and to a lesser extent in Europeans (3.5, at 37.7 kb) and Asians (2.8, at −29.7 kb). F_ST_ at the *CYP2C19* locus was low overall (0.098). CYP2C19*3 was an F_ST_ outlier in Asians (0.293), *CYP2C19* haplotype diversity < = 0.037, p <0.001.

**Conclusions:**

We found some evidence that the slow metabolizing allele CYP2C19*2 is subject to positive selective forces worldwide. Similar evidence was also found for CYP2C19*3 which is frequent only in Asia. F_ST_ is low at the *CYP2C19* locus, suggesting balancing selection overall. The biological factors responsible for these selective pressures are currently unknown. One possible explanation is that early humans were exposed to a ubiquitous novel toxin activated by CYP2C19. The genetic adaptation took place within the last 10,000 years which coincides with the development of systematic agricultural practices.

## Background

The cytochrome P450 enzymes (hereafter referred to as cytochromes) have a wide range of essential biological functions in humans resulting from oxidation of their substrates. CYP2C19 is a key member of the cytochrome family and is responsible for metabolizing a substantial proportion of pharmacologically important compounds [1-8] although it is known to metabolize only a relatively small number of environmental toxins [9,10]. The enzyme is encoded by the *CYP2C19* gene which is situated in the *CYP2C* gene cluster on chromosome 10 where it is in strong linkage disequilibrium with *CYP2C9*[11]. *CYP2C19* exhibits considerable genetic polymorphism giving rise to profound changes in enzyme activity leading to reduced metabolic capacity [12]. This ‘poor metabolizer’ phenotype is common worldwide occurring at 0.02-0.05 in Caucasians and 0.18-0.23 in Asians [13,14]. However the evolutionary mechanisms driving this remarkable allelic diversity which have shaped the drug metabolizing repertoire of modern humans have not previously been investigated.

Early investigations into the genetic basis for the poor metabolizer phenotype revealed a single base pair mutation in exon 5 of *CYP2C19* (19154G/A) which creates an aberrant splice site altering the reading frame of the mRNA and introducing a premature stop codon. This allele, designated CYP2C19*2, results in a non-functional protein [15]. CYP2C19*2 is common in Europe (allele frequency 0.16), Africa (0.14), China (0.26) and Japan (0.28) [16] but does not completely account for the poor metabolizer phenotype in Asia where another poor metabolizing allele CYP2C19*3 is also common (0.08) [17]. CYP2C19*3 is rare in Europeans and Africans (0–0.01) [17-20]. The molecular basis for CYP2C19*3 is a guanine to adenine mutation at position 636 of exon 4 (636G/A) which creates a premature stop codon downstream resulting in a non-functional protein. The prevalence of these slow metabolizing alleles tends to increase moving east so in some parts of East Asia CYP2C19*2 and *3 allele frequencies rise to 0.71 and 0.13 respectively making the slow metabolizer phenotype predominant [21]. CYP2C19 ultra-rapid metabolism is conferred by a gain-of-function allele (*17) (−*806C* > *T*, −3402C > T [22]) which is an important determinant of the rate of metabolism of certain drugs [19]. CYP2C19*17 occurs at a high frequency in Yoruba (0.28), Gambians (0.24), Ethiopians and Swedish (0.18) and is rare in Chinese (0.04) [19,22].

Because of a potential role in metabolizing unknown environmental substances, alleles that cause a change in function of the enzyme would be likely to give an individual a selective advantage or disadvantage in evolutionary terms. For example if the cytochrome is involved in promoting the elimination of a toxic chemical, a gain-of-function allele might confer evolutionary advantage and would thus be positively selected. Conversely if the enzyme catalysed the formation of a toxic compound from an otherwise non-toxic precursor, then alleles conferring low enzyme activity might be selected.

New techniques for identifying such selective pressures on genes in specific populations have been developed leading to significant new insights into human genetic diversity [23]. When a novel allele of a gene initially arises in a population, it is associated with a single haplotype. If this haplotype undergoes a rapid increase in frequency because of positive selection, there is little time for recombination events to break the ancestral links with nearby genetic markers [24]. Hence recently selected alleles tend to display lower haplotypic diversity than do ancient alleles of similar frequency. Thus exploring the length of stretches of homozygosity (extended haplotype homozygosity or EHH) is a way of detecting recent positive selection in the human genome from haplotype structure. EHH is defined as the probability that chromosomes from two randomly chosen individuals are identical from a selected ‘core’ region to a point X. The core is usually selected as a few single nucleotide polymorphisms (SNPs) around a polymorphism that either causes a major functional change in a protein or results in a particular phenotype and the similarity between the chromosomes is measured by the length of homozygosity of genetic markers.

In an extension of this idea, relative extended haplotype homozygosity (REHH) is the ratio of the EHH of the core haplotype compared with the EHH of all other haplotypes on the chromosome. The REHH test compares the length of haplotype identity as a function of frequency around each allele compared to an expected distribution [25]. The degree of haplotype diversity can also be explored graphically using bifurcation plots which are diagrams for visualizing the breakdown of linkage disequilibrium (LD) along haplotypes. These methods are complementary to more conventional population based measures used to examine whether differences in frequencies of markers in specific ethnic groups may have arisen by chance [26].

Recent studies have highlighted the importance of infectious agents such as malaria [23,27] and viral haemorrhagic fever [28,29] in shaping the human genome. Similarly the challenges of adapting to the physical environment have left signals of positive selection in genes related to skin pigmentation [30,31] and development of hair follicles [32]. However there is little evidence to date that potentially harmful chemical substances in the environment have exerted significant evolutionary selective pressure.

In view of the key role of cytochrome P450 enzymes in metabolism of environmental compounds, we investigated the *CYP2C19* locus for signatures of evolutionary selection. We focussed specifically on the common non-functional *CYP2C19* mutations CYP2C19*2 (19154G/A) and *3 (636G/A), measuring their haplotype frequencies and molecular diversity across populations, aiming to identify signals of recent evolutionary selection that might result from exposure to potentially harmful environmental substances.

## Results

### Analysis of extended haplotype homozygosity at the *CYP2C19* locus

**Figure 1:**
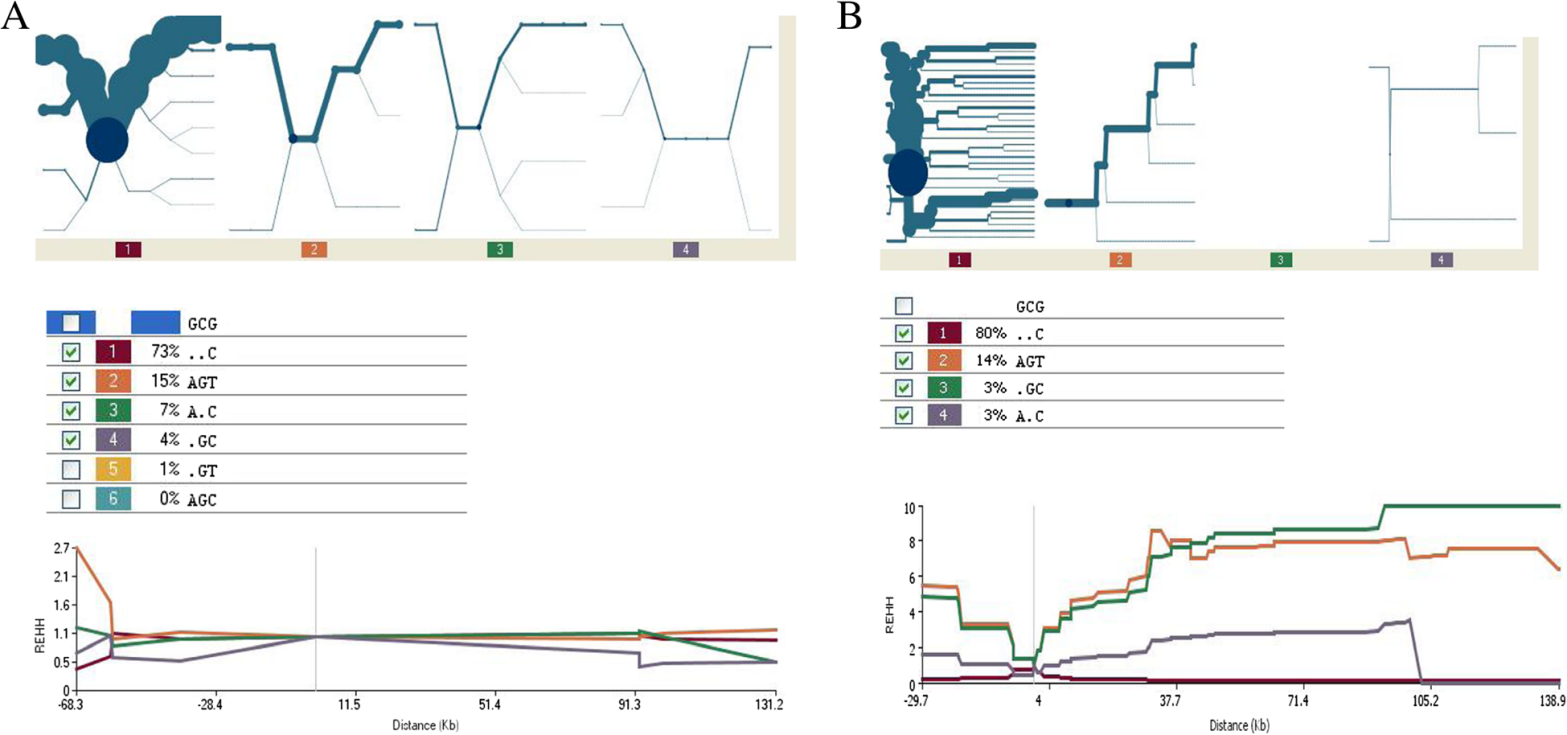
Haplotype bifurcation plots and extended haplotype homozygosity diagrams for *CYP2C19 *slow metabolizing allele (*2) in West Africans. The core for the haplotypes in the first four diagrams is centred at the genomic position of the CYP2C19*2 variant (SNP positions rs4244285, rs4417205 and rs3758580) which is universally present and known to encode a functionally inactive protein. The upper part of each diagram shows a haplotype bifurcation plot representing the degree to which a haplotype is broken down by the emergence of new mutations in the gene. The lower graph shows relative homozygosity for each haplotype (depicted by the coloured lines, described in Figure 2) plotted against genomic position. High values observed some distance from the core indicate extended homozygosity and suggest recent positive evolutionary selection. Common core haplotypes and their frequencies are indicated in Figure 2. **A**. *CYP2C19* haplotype structure in Gambians. The CYP2C19*2 containing haplotype AGT (haplotype 2) is relatively conserved in the bifurcation plot as demonstrated by the single thick line and by relatively few, thinner branches. There is some limited evidence of extended homozygosity. **B**. *CYP2C19* haplotype structure in Yoruba. The CYP2C19*2 containing haplotypes AGT (haplotype 2) and GGC (haplotype 4) both show conservation in the bifurcation plots and extended haplotype homozygosity.

**Figure 2:**
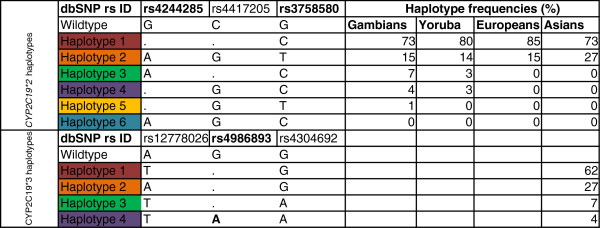
***CYP2C19 *poor metabolism haplotypes and their frequencies in the study populations.** Sweep 1.1 was used to determine haplotypes and extended haplotype homozygosity at the CYP2C19*2 locus from 500 individuals including Gambians and HapMap populations. CYP2C19*3 was found only in the Asian population.

Bifurcation plots for the common loss-of-function allele CYP2C19*2 in the merged genotypic data from Gambians are shown in Figure 1A. The haplotypes for CYP2C19*2/*3-containing alleles are described in Figure 2.

The bifurcation plot for the haplotype carrying CYP2C19*2 (AGT) suggests some evidence of positive selection, shown by the predominance of thick branches. This haplotype, present at a frequency of 0.15, had an REHH of 2.7 at −68.3 kb from the core region which is supportive of the suggestion of positive selection for CYP2C19*2. Since our genotyping data did not extend further than −68.3 kb from the core haplotype it was not possible to examine REHH over longer stretches of the genome in the Gambian study group.

We therefore examined the HapMap data for the Yoruba to obtain more extensive genomic data from people living in a nearby area of West Africa (Figure 1B). The same CYP2C19*2 AGT-haplotype was present in the Yoruba at a similar frequency to Gambians (0.14) and displayed extended homozygosity evidenced by conservation on the bifurcation plot and an REHH of 8.3 at 133.3 kb from the core. This implies that the slow metabolism allele can confer an evolutionary advantage and confirms the initial findings in Gambians. The magnitude of the REHH for CYP2C19*2 in Yoruba is similar to that observed for G6PD-202A (glucose-6-phosphate dehydrogenase deficiency) which is thought to confer a 50% reduction in risk of malaria [23,33].

**Figure 3:**
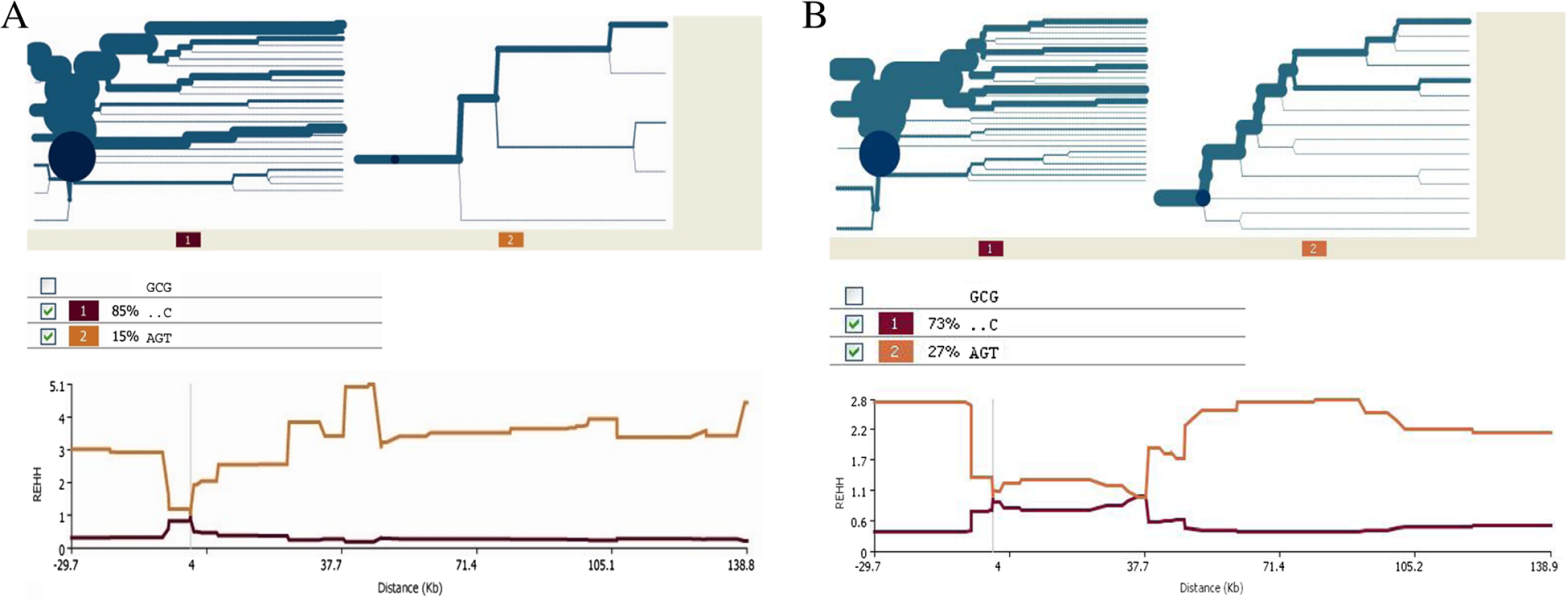
Haplotype bifurcation plots and extended haplotype homozygosity diagrams for *CYP2C19 *slow metabolizing allele (*2) in Europeans and Asians. A. *CYP2C19* haplotype structure in Europeans. CYP2C19*2 containing AGT (haplotype 2) shows a branching pattern and the level of extended homozygosity is lower than observed in Yoruba. **B**. CYP2C19*2 haplotype structure in Japanese and Han Chinese. The CYP2C19*2-containing haplotype AGT (haplotype 2) has a branching structure and REHH values similar to those in Europeans.

Analysis of CYP2C19*2 for positive selection in Europeans showed similar patterns to those observed in Yoruba (Figure 3A). The AGT haplotype occurred at a similar frequency (0.15) with an REHH value of 3.5 at 37.7 kb from the core. The CYP2C19*2 bifurcation plot for Europeans showed some breakdown of the haplotype depicted by the thinning of the branch indicating the emergence of a new mutation. These results provide some evidence for CYP2C19*2 selection in Europeans, however the effect appears to be less strong than that observed in West Africans.

**Figure 4:**
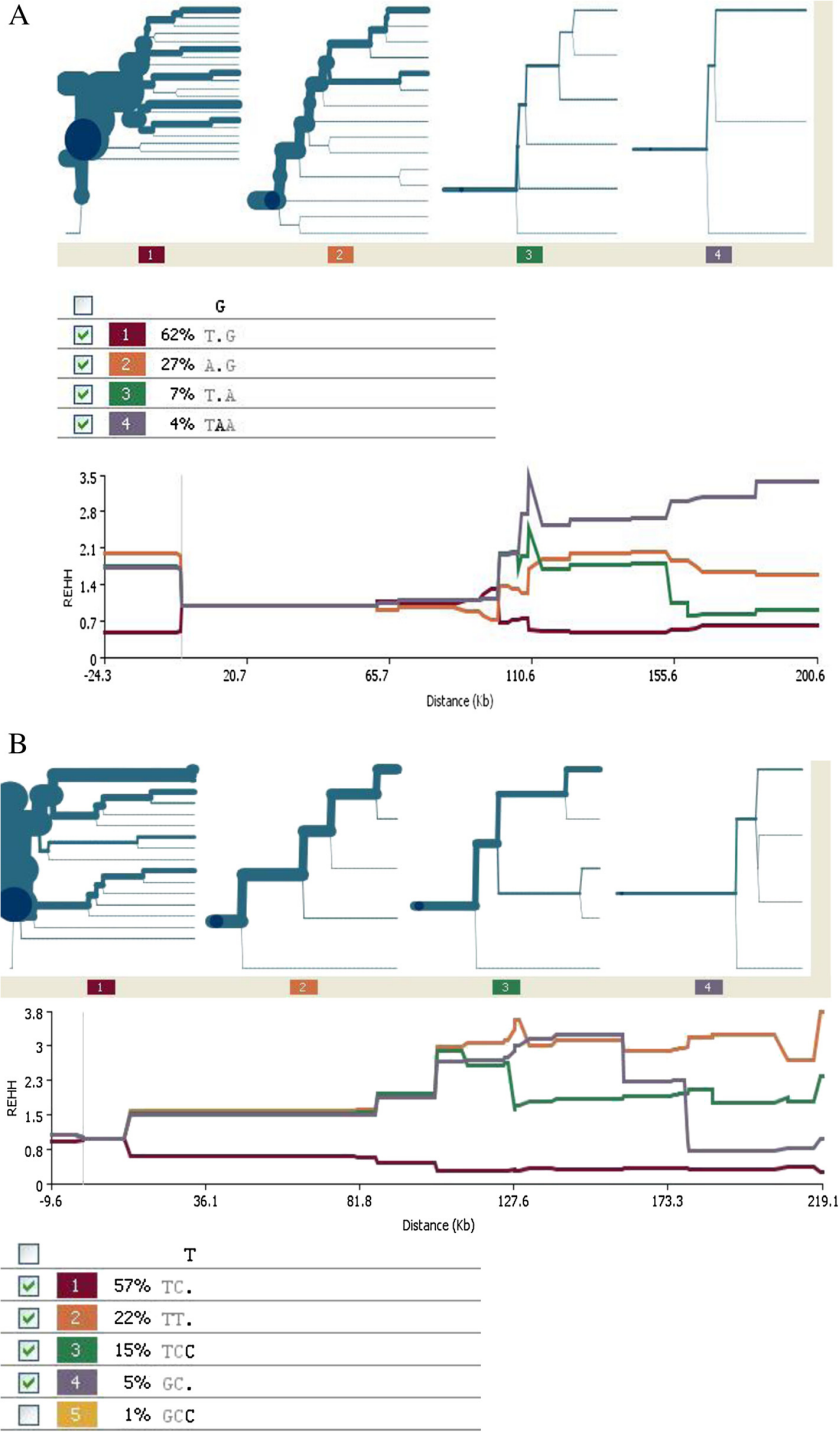
Haplotype bifurcation plots and extended haplotype homozygosity diagrams for the CYP2C19*3 slow metabolizing allele in Asians and the gain-**of**-function allele CYP2C19*17 in Europeans. The plot is centred on the common, slow metabolizing allele which is common in Asians CYP2C19*3 at SNP positions rs12778026, rs4986893 (CYP2C19*3) and rs4304692 and EHH was measured away from the core in Asians. The high activity allele core region in Europeans is focused on SNP positions rs11568732, rs12248560 (CYP2C19*17) and rs4986894 and the haplotype extension measured from the core locus. **A**. CYP2C19*3 haplotype structure in Japanese and Han Chinese. The CYP2C19*3 mutation is carried on haplotype TAA (haplotype 4) which shows some evidence of extended homozygosity. **B**. CYP2C19*17 haplotype structure in Europeans. The CYP2C19*17 variant is carried on haplotype TTT (haplotype 4) that extends along the genome without a breakdown of its homozygosity showing evidence of selection in Europeans. The haplotype table below the figure describes the CYP2C19*17-containing haplotypes in the European population.

Japanese and Han Chinese had the highest CYP2C19*2 AGT frequency at 0.27 and the lowest REHH (2.8 at −29.7 kb from the core). The bifurcation plot for the Japanese and Han Chinese showed clear breakdown of haplotype homozygosity arising from development of a new mutation, depicted by thinning of the branch (Figure 3B). This suggests that CYP2C19*2 is not subject to recent positive selection in this population. However in contrast CYP2C19*3 (rs4986893), which also codes for an inactive enzyme only found in Asia shows some evidence of extended homozygosity (Figure 4A).

Interestingly, analysis for extended haplotype homozygosity around the CYP2C19*17 allele in Gambians, Yoruba and Asians showed no evidence of selection. Only Europeans demonstrated a signature of selection at the CYP2C19*17 locus where the TTT-haplotype frequency is high at 22% and its bifurcation plot does not breakdown the haplotype homozygosity despite the generation of new SNPs from recombination events (Figure 4B).

### Allele frequency differences for *CYP2C19* across populations from West Africa, Europe and Asia

F_ST_ at the *CYP2C19* locus was low overall across the three populations implying balancing selective forces (Table 1). The F_ST_ for CYP2C19*2 (rs4244285) was 0.016 implying that only 1.6% of human genetic diversity at CYP2C19*2 is a result of genetic differentiation among the four populations studied. The CYP2C19*3 allele in Japanese and Han Chinese had a high F_ST_ implying unopposed positive selection in this population. Pairwise F_ST_ values are given in Additional file 1: Table S1. Coalescent simulations of the genotypic data showed significant haplotype diversity < = 0.037 (p <0.001) for the *CYP2C* alleles screened in the Gambians and HapMap populations.

**Table 1:** **F**_**ST **_**measure of population differentiation and minor allele frequencies at *CYP2C19/9 *** **locus in Gambians and in the three HapMap populations**

	**F**_**ST **_**per locus**	**Allele frequency**			
***CYP2C19***		**Gambians**	**Europeans**	**Asians**	**Yoruba**
rs4244285 (CYP2C19*2)	0.016	0.23	0.15	0.27	0.15
rs12248560 (CYP2C19*17)	0.080	0.23	0.21	0.01	0.28
rs4986893 (CYP2C19*3)	0.295	0	0	0.53	0
rs7067866	0.078	0.39	0.57	0.61	0.29
rs4417205	0.011	0.18	0.15	0.27	0.18
rs4986894	0.015	0.18	0.16	0.27	0.14
***CYP2C9***					
rs1799853 (CYP2C9*2)	0.05	0.03	0.09	0	0
Overall	0.098				

FSTAT 2.9.3 was used to estimate F_ST_. The genotype data for the individual SNPs of the HapMap populations were retrieved from NCBI dbSNP BUILD 131.

## Discussion

We found some evidence that non-functional haplotypes of *CYP2C19* (CYP2C19*2, CYP2C19*3) may be subject to selective forces in recent human evolution. Slow metabolizing haplotypes of *CYP2C19* showed extended homozygosity which results from recent positive selection. We inferred this initially in the Gambian population for CYP2C19*2 and these findings were confirmed by analysis of genotypic data in Yoruba, from a nearby region of West Africa. We observed a similar pattern of haplotype homozygosity in Europeans, however the CYP2C19*2 EHH is more striking in Yoruba suggesting stronger positive selection in the West African population. Interestingly the fast metabolizing CYP2C19*17 was also under positive selection in Europeans. The REHH statistic observed for CYP2C19*2 in Yoruba (REHH 8.3) is of a similar magnitude to that seen in the same ethnic group for G6PD [23]. Selection pressure on G6PD is thought to be due to protection from malaria which is a major cause of death in childhood in Africa.

CYP2C19*2 accounts for the poor metabolizing phenotype in Europeans and in people of African descent, whilst CYP2C19*3 contributes to the poor metabolizer phenotype only in Asia. In Asian populations we found some evidence that CYP2C19*3 is also positively selected. This suggests that the CYP2C19 poor metabolism phenotype confers an evolutionary advantage in Asia in addition to Africa and Europe even though some alleles responsible for that phenotype differ between the three continents.

All F_ST_ values estimated for the *CYP2C19* SNPs are lower than the average F_ST_ values for autosomal SNPs in worldwide human populations which is approximately 0.123. The *CYP2C19* F_ST_ values are consistent with the low F_ST_ observed for many of the genes involved in immunity such as the major histocompatibility complex (MHC) and beta-globin gene which are under balancing selection [34]. The low F_ST_ is an indication that balancing or species-wide directional selection has taken place at the *CYP2C19* locus, in contrast to the force of geographically-restricted directional selection that leads to high F_ST_ values. Thus one would infer from the long extended haplotype and low F_ST_ for the slow-metabolizer CYP2C19*2 allele that CYP2C19*2 is still evolving and has not yet reached fixation.

Investigations of evolutionary selection of alleles in the *CYP2C* cluster in the past have been relatively limited. Vormfelde and colleagues analysed selection signals in *CYP2C9*[1] and showed that the low activity allele CYP2C9*3 appeared to be under positive selection. There is a high degree of Linkage Disequilibrium (LD) in the *CYP2C* cluster so it is possible that the effects we saw on *CYP2C19* might reflect selection of alleles of *CYP2C9* and *CYP2C8*[11] however the extended haplotypes that we found did not stretch beyond the *CYP2C19* genomic region. CYP2C9*3 was not screened in this project as the variant has not been found in sub Saharan Africa.

Our finding of positive selection for CYP2C19*2 poor functional variant worldwide is in accordance with the recent findings by Pimenoff and colleagues who used a similar technique (EHH) and haplotype structure to describe selection pressure on poor activity variants CYP2C19*2 and CYP2C9*3 in Europeans [35]. Our study further highlights CYP2C19*3 to be under selective pressure in Asians and the high activity CYP2C19*17 in Europeans. Poor activity alleles especially are favoured in recent human evolution, being selected in Africa, Europe and Asia. A further analysis of cytochrome gene evolutionary selection in the indigenous American populations would give interesting additional information.

### Time scale of the evolutionary processes

The population selection forces acting on *CYP2C19* alleles are likely to have been operating over the last 10,000 years [23,36]. An allele under positive selection rapidly rises in prevalence such that recombination does not substantially break down the association with alleles at nearby loci on the ancestral chromosome. This characteristic of an allele under positive selection is short-lived because recombination rapidly breaks down the long-range haplotypes.

### Possible biological factors causing selective pressure on inactive *CYP2C19* alleles

It is interesting to speculate what biological forces are responsible for evolutionary selection of inactive alleles of *CYP2C19*. The period of selection would be likely to correspond with the end of the most recent glacial age and beginning of the Mesolithic era (Middle Stone Age), from 8500 BC to 4000 BC. Over this period there is substantial archaeological evidence of developing systematic agricultural practices in Asia and the Middle East in contrast to the previous hunting and gathering lifestyle of early humans [37].

The storage of grain and tubers from one growing season to another, which is essential for effective agriculture, would have exposed early humans to a range of novel toxins derived from fungi growing on stored foodstuffs. Activation of mycotoxins by cytochrome P450 enzymes is well-described [38,39] and these toxins remain a major health problem in Sub Saharan Africa to the present day. Recently, CYP2C19 has been implicated in the metabolism of the cytotoxic mycotoxin enniatin B, a very stable secondary fungal metabolite of *Fusarium* strains that contaminate cereals and grains [40].

In addition to mycotoxins, other classes of harmful environmental compounds may have been encountered for the first time as a result of developing agricultural practices. For example aryl hydrocarbons formed from incomplete combustion of carbon are highly toxic when partially activated by CYP2C19 and might be produced by the use of fire to clear land for crops [10].

Earlier speculations by Nebert suggested that rapid evolution of drug metabolizing enzyme (DME) genes and receptor genes occurred as a result of the interaction between animals as they moved on to land and the plants that they encountered there. An explosion of new animal genes in the *CYP2* family occurred nearly 400 million years ago with more than 50 gene duplication events [41]. Nelson and colleagues argue that *CYP* evolution started much earlier >600 million years ago at the same gene locus referred to as the cytochrome P450 genesis locus from where all CYP clans or families emerged [42]. The DME genes then further evolved to produce polymorphic enzymes that may have functions diminished or enhanced as a consequence of selection of alleles perhaps in response to diet [43]. Such factors may give rise to the selective pressures that maintain the high frequency of the poor metabolizing alleles of CYP2C19*2/*3[42-44].

The physiological functions of CYP2C19, particularly in the synthesis of steroid hormones, could also potentially be important in increasing survival fitness [45]. Gomes and colleagues in 2009 implicated CYP2C19/CYP3A4 in 21-hydroxylation of progesterone in individuals with 21-hydroxylase deficiency thus affecting levels of mineralocorticoids [46]. A recent study has linked CYP2C19*2 rs4244285 with the regulation of blood pressure [47]. CYP2C19 may also influence arachidonic acid metabolism predisposing to peptic ulcer disease [48,49] and vascular disease [50]. These functions might also influence the interaction with environmental pathogens although the mechanism by which the resulting selective force might operate and the strength of the selective pressure that would result is not clear.

Whilst the mechanism for positive selection of individuals with poor CYP2C19 activity remains unknown, the evidence for such selection in *CYP2C19* seems to be persuasive. The magnitude of the evolutionary pressure appears to be similar to that exerted on the human genome by infectious diseases. These environmental and/or physiological forces that shaped the cytochrome repertoire of modern humans have important consequences for drug metabolism in the present day [19].

The selective forces that we observed are temporally linked to the development of systematic agricultural practices by early humans and CYP2C physiological functions although it is not possible to infer a direct causal association from our data. It would be useful to extend these studies to other populations where different agricultural practices might have exposed humans to a different range of potentially harmful environmental chemical compounds. Similar signatures of selection might be found for other cytochrome P450 enzymes that activate known mycotoxins [10,38]. In addition, if the selective pressure were causally related to the use of agriculture then we would expect to see low levels of extended homozygosity in isolated populations that continued to maintain a ‘hunter gatherer’ lifestyle.

Further work could examine the co-evolution of cytochromes with other elements of the metabolic pathways involved in detoxification of xenobiotic substances and metabolism of drugs. Some speculative studies indicate that considerable synergisms may exist between certain isoforms of cytochromes and transporter molecules that regulate influx of their substrates into cells [51]. If this is the case then patterns of inheritance of phenotype are likely to be highly complex since drug transporter status may have a permissive effect on either fast or slow cytochrome metabolism. Thus, in the absence of linkage disequilibrium, determining genotype at either locus independently would not reliably predict metabolic phenotype.

Inclusion of longer stretches of the genome in increased numbers of individuals could identify long range haplotypes in cytochrome gene clusters which have been positively selected. These haplotypes delimited by rapidly decaying LD would identify biologically important combinations of alleles across the gene cluster [11]. Such haplotypes could be functionally more important – for example in drug metabolism – than alleles of the individual genes.

Since we have identified some evidence of global evolutionary selective forces in favour of alleles of *CYP2C19* that have low activity in the metabolic transformation of xenobiotic compounds it seems reasonable to suggest that some environmental agent would be responsible for exerting this pressure and shaping the cytochrome profile of modern humans. This agent could be a known substrate for CYP2C19 which has a metabolite with unrecognised toxic effects or alternatively a known toxin which is activated by the enzyme by a novel molecular mechanism. Either of these hypotheses might provide a fruitful starting point for further biochemical and toxicological research. Such studies might cast further light on human evolution and could potentially identify substances not previously known to be toxic.

## Conclusions

Many forces shape the topography of the human genome and strong natural selection resulting from infectious diseases is well–recognised. Here we show that environmental chemicals could also exert a similar effect on human evolution.

We speculate that a ubiquitous environmental compound may be rendered toxic by the activity of CYP2C19 and thus early humans with poor CYP2C19 activity had a survival advantage. This genetic adaptation took place within the last 10,000 years which coincides with the development of systematic agricultural practices. These evolutionary forces, which are of a similar magnitude to those exerted by infectious diseases, could have arisen from exposure to a novel toxin perhaps arising from stored foodstuffs. Selective pressure from this toxin may have driven allelic differentiation at the *CYP2C19* locus and hence strongly influenced the drug metabolizing profile of modern humans.

## Methods

### Study participants

Eighty-five Gambian blood donors from Sukuta and 42 from Brikama (Western Region), Njaba Kunda and Farafenni (Northern Region) gave informed consent for genetic screening and analysis. The subjects from Sukuta participated in an investigation of nucleotide diversity of the TNF gene region [52] and the remainder adult cohort participated in a randomised controlled trial of chlorproguanil-dapsone/co-artemether for uncomplicated malaria [53] (Clinical Trials Identifier: NCT00118794).

Venous blood was collected in EDTA tubes and deoxyribonucleic acid (DNA) extracted using Nucleon kits BACC3 (Tepnel Life Sciences, Britain) according to the manufacturer’s protocol. The DNA was quantified using Picogreen (Molecular Probes®) and the NanoDrop 1000™ spectrophotometer (Thermo Scientific) and stored at −20°C until required.

Metabolic phenotype for *CYP2C19* had been previously characterized in fine detail in the adult study group and all common fast and slow metabolizing alleles identified [19]. TaqMan^®^ drug metabolizing assay mixes for SNP genotyping were used to screen functional polymorphisms and amplification refractory mutation system PCR to genotype promoter and intronic polymorphisms in *CYP2C19* and *CYP2C9* in all the subjects. The 13 SNPs IDs genotyped were:

1. *CYP2C19*: rs7067866, rs11568729, rs4417205, rs4986894, rs3758580 (mRNA990 *2A*2B), rs4986893 (mRNA636 *3), rs17884712 (mRNA431 *9), rs4244285 (mRNA681 *2), rs12248560 (*17) and

2. *CYP2C9*: rs1799853 (*2), rs7900194 (*8), rs2256871 (*9), rs28371686 (*11).

Genotypic data from the two Gambian study groups were pooled to give a sample size of 127 subjects.

Ethical approval was obtained from the Medical Research Council (MRC) Scientific Coordinating Committee and the MRC/Gambia Government Joint Ethics Committee (L2005.80, SCC No. 981 11th January 2005). Consent was written in English and explained in the local language of the subjects by an interpreter and the response was documented in English on the consent form.

### Bifurcation plot and relative extended haplotype homozygosity diagram construction

First we constructed relative extended haplotype homozygosity (REHH) and haplotype bifurcation plots to look for positive selection of CYP2C19*2 in the Gambian population using SWEEP [54]. CYP2C19*3 was not present in any Gambian participant. Missing allelic data were filled in using fastPHASE version 2.3 [55]. We then sought to validate signatures of positive selection observed for Gambian haplotypes using 120 chromosomes from HapMap population panels initially West African Yoruba from Nigeria and then Europeans from Utah and Asians of Japanese and Han Chinese origin: International HapMap Project, HapMap Data Rel 24/phaseII Nov8, on NCBI B36 assembly, dbSNP b126 [16]. These data were analysed for haplotype breakdown at the ***CYP2C19*** * ***2 *** **locus** with the **core haplotype rs4244285**-**rs4417205**-**rs3758580** and for ***CYP2C19*** * ***3*** where it was present (**rs12778026**-**rs4986893**-**rs4304692**). We generated REHH and bifurcation plots from rs7067866 (96501916) to rs9332198 (NCBI build35 96731487) for the Yoruba, to rs1934967 (96731416) for CEU and to rs9332198 (96731487) for the Han Chinese. These plots were then examined for signals of selective pressure.

The F_ST_ statistic was calculated to summarise allele frequency differentiation between populations using FSTAT 2.9.3 [56]. Haplotype diversity was estimated by coalescent simulations of the genotypic data in DnaSP 5.10.01 after 1000 replicates [57]. The input data consisted of a sample size of 500, simulations given theta value 12.5 and a pseudorandom number seed 2999311.

## Abbreviations

CYP2C9: Cytochrome P450 2C9; CYP2C19: Cytochrome P450 2C19; EHH: Extended haplotype homozygosity; LD: Linkage disequilibrium; REHH: Relative extended haplotype homozygosity; SNP: Single nucleotide polymorphism.

## Competing interests

The authors declare that they have no competing interests.

## Authors’ contributions

RTW, REJ and AW contributed to study conception and design. REJ and FSJ participated in the acquisition of data. RTW, REJ and AW took part in data analysis. REJ and RTW drafted the manuscript. REJ, AW, SOS, KJL and RTW interpreted the results. All authors have read and approved the final version of the manuscript.

## Supplementary Material

Additional file 1: Table S1 F_ST_ for *CYP2C19* alleles across Gambians, Europeans, Japanese and Han Chinese and Yoruba. Click here for file
