# Supplementary material for: Inactive alleles of cytochrome P450 2C19 may be positively selected in human evolution
Source: BMC Evol Biol. 2014 Apr 1;14:71. doi: 10.1186/1471-2148-14-71 (PMC4036532; doi:10.1186/1471-2148-14-71)
Supplement: Additional file 1: Table S1 — FST for CYP2C19 alleles across Gambians, Europeans, Japanese and Han Chinese and Yoruba. [file 1471-2148-14-71-S1.doc]

Additional file 1: Table S1**.** **F_ST_ for *CYP2C19* alleles across Gambians, Europeans, Japanese and Han Chinese and Yoruba.**

|  | **Europeans** | **Han Chinese** | **Yoruba** |
| --- | --- | --- | --- |
| **Gambians** | 0.1214 | 0.1635 | 0.1112 |
| **Europeans** |  | 0.0350 | 0.0368 |
| **Han Chinese** |  |  | 0.0866 |

Pairwise F_ST_ was estimated using FSTAT 2.9.3.
